# Insulin reduces endoplasmic reticulum stress‐induced apoptosis by decreasing mitochondrial hyperpolarization and caspase‐12 in INS‐1 pancreatic β‐cells

**DOI:** 10.14814/phy2.16106

**Published:** 2024-06-17

**Authors:** Nanako Murata, Kana Nishimura, Naoki Harada, Tomoya Kitakaze, Eiji Yoshihara, Hiroshi Inui, Ryoichi Yamaji

**Affiliations:** ^1^ Department of Applied Biological Chemistry, Graduate School of Agriculture Osaka Metropolitan University Sakai Osaka Japan; ^2^ Division of Applied Life Sciences, Graduate School of Life and Environmental Sciences Osaka Prefecture University Sakai Osaka Japan; ^3^ The Lundquist Institute for Biomedical Innovation at Harbor‐UCLA Medical Center Torrance California USA; ^4^ David Geffen School of Medicine at University of California Los Angeles Los Angeles California USA; ^5^ Department of Health and Nutrition Otemae University Osaka Japan; ^6^ Center for Research and Development of Bioresources Osaka Metropolitan University Sakai Osaka Japan

**Keywords:** autocrine and paracrine actions of insulin, caspase‐12, cytochrome *c*, endoplasmic reticulum (ER) stress, mitochondrial hyperactivation, pancreatic β‐cells

## Abstract

Pancreatic β‐cell mass is a critical determinant of insulin secretion. Severe endoplasmic reticulum (ER) stress causes β‐cell apoptosis; however, the mechanisms of progression and suppression are not yet fully understood. Here, we report that the autocrine/paracrine function of insulin reduces ER stress‐induced β‐cell apoptosis. Insulin reduced the ER‐stress inducer tunicamycin‐ and thapsigargin‐induced cell viability loss due to apoptosis in INS‐1 β‐cells. Moreover, the effect of insulin was greater than that of insulin‐like growth factor‐1 at physiologically relevant concentrations. Insulin did not attenuate the ER stress‐induced increase in unfolded protein response genes. ER stress did not induce cytochrome *c* release from mitochondria. Mitochondrial hyperpolarization was induced by ER stress and prevented by insulin. The protonophore/mitochondrial oxidative phosphorylation uncoupler, but not the antioxidants *N*‐acetylcysteine and α‐tocopherol, exhibited potential cytoprotection during ER stress. Both procaspase‐12 and cleaved caspase‐12 levels increased under ER stress. The caspase‐12 inhibitor Z‐ATAD‐FMK decreased ER stress‐induced apoptosis. Caspase‐12 overexpression reduced cell viability, which was diminished in the presence of insulin. Insulin decreased caspase‐12 levels at the post‐translational stages. These results demonstrate that insulin protects against ER stress‐induced β‐cell apoptosis in this cell line. Furthermore, mitochondrial hyperpolarization and increased caspase‐12 levels are involved in ER stress‐induced and insulin‐suppressed β‐cell apoptosis.

## 1 INTRODUCTION

Pancreatic β‐cells contribute to maintain blood glucose homeostasis by secreting insulin in response to an elevation in postprandial blood glucose concentrations, which leads to glucose uptake in the peripheral tissues (DeFronzo, 2004). Individual β‐cell function and total β‐cell mass are crucial for circulating concentrations of insulin. Therefore, the deficiency of insulin caused by impaired quality and quantity of β‐cells is associated with the pathogenesis of type 2 diabetes mellitus (T2DM) (Butler et al., 2003; Meier & Bonadonna, 2013; Weir et al., 2020). Moreover, β‐cell dysfunction is suggested to precede β‐cell loss (Cohrs et al., 2020) which is due to an increase in apoptotic cell death and is frequently observed in patients with T2DM (Butler et al., 2003; Rhodes, 2005). However, detailed mechanisms underlying the causality between these two events have not been fully elucidated.

Persistent endoplasmic reticulum (ER) stress occurs in pancreatic β‐cells due to a large amount of insulin production, and this stress is higher than in other cells (Tsuchiya et al., 2018). ER stress induces the unfolded protein response which activates IRE1α/XBP‐1, PERK/ATF4, and ATF6 signaling to reduce and adapt to the stress (Hetz, 2012). The expression of C/EBP homologous protein (CHOP), a transcription factor that promotes ER stress‐induced apoptosis (Song et al., 2008), is induced by ATF4 and ATF6. In addition to CHOP, BIP/GRP78, ATF4, and spliced XBP1 levels increase due to the unfolded protein response and are recognized as markers of ER stress (Cao & Kaufman, 2014). Tunicamycin and thapsigargin are commonly used ER stress inducers that dysregulate ER homeostasis by inhibiting *N*‐glycosylation and blocking ER Ca^2+^ ATPase, respectively (Oslowski & Urano, 2011). However, chronically high demand for insulin biosynthesis burdens the ER capacity and induces apoptosis in β‐cells (Hetz, 2012; Xu et al., 2005). Although it is proposed that ameliorating ER stress could prevent β‐cell apoptosis, the mechanism underlying progression and suppression of ER stress‐induced apoptosis are not fully understood.

Mitochondria play key roles not only in energy production but also in apoptotic cell death (i.e., through the mitochondrial intrinsic pathway) (Bock & Tait, 2020). In the latter process, the loss of mitochondrial membrane potential increases the release of cytochrome *c* from the mitochondrial inner membrane, which sequentially activates the downstream caspases (Bock & Tait, 2020). Caspase‐12 has been proposed to mediate ER stress‐induced apoptosis in a mitochondria‐independent manner (i.e., the ER intrinsic pathway) (Lamkanfi et al., 2004; Nakagawa et al., 2000; Rao et al., 2002; Szegezdi et al., 2003).

Insulin not only acts as a regulator of glucose homeostasis but also as an anabolic hormone. In pancreatic β‐cells, insulin signaling is essential for β‐cell mass formation and maintenance by regulating cell proliferation and apoptosis (Johnson et al., 2006; Leibiger et al., 2008; Okada et al., 2007; Otani et al., 2004). However, whether insulin inhibits ER stress‐induced β‐cell apoptosis remains unclear. Here, we show that insulin slightly increased the unfolded protein response and reduced ER stress‐induced mitochondrial hyperpolarization, caspase‐12 activation, and apoptosis independent of the mitochondrial apoptosis pathway in β‐cells.

## 2 MATERIALS AND METHODS

### 2.1 Cell culture

INS‐1 rat pancreatic β‐cells (RRID:CVCL_0352) (Asfari et al., 1992) were cultured in RPMI 1640 medium supplemented with 10% fetal bovine serum (FBS), 11.1 mM d‐glucose, 10 mM HEPES, 1 mM sodium pyruvate, 50 μM 2‐mercaptoethanol, 100 U/mL penicillin, and 100 μg/mL streptomycin. The cells were maintained at 37°C in a 5% CO_2_/95% air atmosphere at 98% humidity.

### 2.2 Cell viability assay

For cell viability assays using tunicamycin (202–08241, Wako Pure Chemical Industries, Osaka, Japan) and thapsigargin (T‐9033, Sigma‐Aldrich, St. Louis, MO, USA), cells were seeded onto 48‐well plates at a density of 5.0 × 10^4^ cells/well and incubated for 24 h. The cells were preincubated with an inhibitor or antioxidants (30–60 min, Z‐ATAD‐FMK, FMK013, R&D Systems, Minneapolis, MN, USA; *N*‐acetyl‐l‐cysteine, 017–05131, Wako Pure Chemical Industries; α‐tocopherol, 341–14, Nacalai Tesque, Kyoto, Japan) and then incubated in the presence or absence of 3 μM tunicamycin and 30 nM thapsigargin for 48 h or 5 mM alloxan monohydrate (A7413, Sigma‐Aldrich) for 24 h with 1 μM insulin (093–02973, Wako Pure Chemical Industries) or IGF‐1 (100‐11R3, Peprotech, Rocky Hill, NJ, USA). We constructed p3 × FLAG‐caspase‐12‐Myc vector and p3 × FLAG‐EGFP‐Myc vectors, expressing N‐terminal FLAG‐tagged and C‐terminal Myc‐tagged mouse caspase‐12 and EGFP, respectively. INS‐1 cells in a 96 well plate were reverse‐transfected with the p3 × FLAG‐caspase‐12‐Myc vector or mock vector (p3 × FLAG‐Myc‐CMV26) using Lipofectamine 3000 (Thermo Fisher Scientific, Waltham, MA, USA) and incubated for 48 h. The medium was replaced with fresh medium containing 5% AlamarBlue (BUF012B, Bio‐Rad, Hercules, CA, USA), followed by incubation for an additional 4 h. Excitation wavelength of 544 nm and emission wavelength of 590 nm was used to measure the fluorescence using a Fluoroscan Ascent FL (Labsystems, Helsinki, Finland).

### 2.3 Subcellular fractionation

Subcellular fractionation was performed as previously described (Harada et al., 2018). Briefly, INS‐1 cells were washed twice and harvested using cellular fractionation buffer (20 mM HEPES‐NaOH, pH 7.5, 250 mM sucrose, 1 mM EDTA, 1 mM sodium orthovanadate, and 10 mM sodium fluoride). The cells were resuspended in the cellular fractionation buffer containing 10 μg/mL leupeptin, 1 μg/aprotinin, 1 mM phenylmethylsulfonyl fluoride, and 1 mM DTT and homogenized using a microhomogenizer with power tool (PT‐α; ISO, Kanagawa, Japan). The homogenate (whole cell lysate) was separated by centrifugation to obtain a nuclear pellet (500 × g for 5 min), mitochondrial pellet (10,000 × g for 20 min), and cytosolic supernatant (100,000 × g for 60 min) as fractions. Each fraction was analyzed by western blotting using a fixed amount of protein.

### 2.4 Western blotting

INS‐1 cells were preincubated with inhibitors (30–60 min) and cultured in the presence and absence of an ER stress inducer and insulin. INS‐1 cells were reverse‐transfected with the p3 × FLAG‐caspase‐12‐Myc vector or p3 × FLAG‐EGFP‐Myc vector using Lipofectamine 3000 and cultured for 48 h in the presence and absence of insulin. The cells were washed twice with TBS, harvested, and sonicated in IP buffer (50 mM Tris–HCl, pH 7.5, 150 mM NaCl, 0.5% Nonidet P‐40, 10 mM sodium pyrophosphate, 1 mM sodium orthovanadate, 10 mM sodium fluoride, 10 mM sodium molybdate, 2 mM EDTA, 1 mM phenylmethylsulfonyl fluoride, 10 μg/mL leupeptin, 1 μg/mL aprotinin, and 1 mM dithiothreitol). The lysate was centrifuged at 20,000 × g for 10 min, and the supernatant was subjected to SDS‐PAGE using a fixed amount of protein. Western blotting was performed using polyclonal rabbit anti‐caspase‐12 (1/1000, 3282–100, Biovision, Waltham, MA, USA; RRID:AB_2243910), polyclonal rabbit anti‐caspase‐9 (1/3000, #9502, Cell Signaling Technology; RRID:AB_2068621), polyclonal rabbit anti‐cleaved caspase‐3 (Asp175) and polyclonal rabbit anti‐caspase‐3 antibodies (1/3000 for both #9661 and #9662, Cell Signaling Technology, Danvers, MA, USA; RRID:AB_2341188 and RRID:AB_331439), monoclonal mouse anti‐Cox IV antibody (1/5000, 4D11‐B3‐E8, Cell Signaling Technology; RRID:AB_2797784), polyclonal rabbit anti‐triosephosphate isomerase antibody (Yamaji et al., 2003), monoclonal mouse anti‐cytochrome *c* (1/1000, 7H8, Santa Cruz Biotechnology, Santa Cruz, CA, USA; RRID:AB_627383), monoclonal mouse anti‐α‐tubulin (1/5000, DM1A, Santa Cruz Biotechnology; RRID:AB_628412), and monoclonal mouse anti‐FLAG antibody (1/5000, M2, Sigma‐Aldrich; RRID:AB_259529). Post reaction with horseradish peroxidase‐conjugated goat anti‐rabbit or goat anti‐mouse antibodies (1/5000, *Bio‐Rad*; RRID:AB_11125142 and RRID:AB_11125547, respectively), immunoreactive bands were developed as previously described (Harada et al., 2012). Band intensities were determined using Image J software version 1.53e (National Institutes of Health, Bethesda, MD, USA; RRID:SCR_003070).

### 2.5 Immunofluorescence microscopy

Cells that had been incubated with 1 μM insulin and 3 μM tunicamycin for 12 h were washed once with culture medium without FBS. Then, the cells were incubated with culture medium without FBS in the presence of 100 nM Hoechest 33342 (346–07951, Dojindo, Kumamoto, Japan) and 1 μM JC‐1 (22200, AAT Bioquest, Sunnyvale, CA, USA) or 500 nM rhodamine 123 (R8004, Sigma‐Aldrich) for 30 min. The protonophore/mitochondrial oxidative phosphorylation uncoupler FCCP (20 μM, C3463, Tokyo Chemical Industry, Tokyo, Japan) was used as a control for mitochondrial membrane depolarization. After removal of the medium, cells were washed twice and re‐infused with Hanks' balanced salt solution, followed by observation using a fluorescence microscope (×20, Ex/Em: 360/460 nm for DAPI (D1306, Thermo Fisher Scientific), 470/525 nm for JC‐1 monomer or rhodamine 123, 545/605 nm for JC‐1 aggregates, BZ‐X810, Keyence, Osaka, Japan). Fluorescence from JC‐1 or Rhodamine 123 was validated using a negative control that did not contain a fluorescent dye. The fluorescence intensity observed under the same conditions was analyzed using Image‐Pro Premier image analysis software (Media Cybernetics, Silver Spring, MD, USA; RRID:SCR_016497).

### 2.6 DNA ladder

INS‐1 cells that were seeded onto a 12 well plate at a density of 2.5 × 10^5^ cells/well were incubated for 24 h. The cells were further incubated in the presence or absence of 1 μM insulin and 3 μM tunicamycin for 48 h. DNA ladder assay was performed as described previously (Harada et al., 2015).

### 2.7 Quantitative RT‐PCR

Total RNA was isolated using Sepasol‐RNA I Super G reagent (09379–55, Nacalai Tesque). After treatment with DNase I (314–09071, Nippon Gene, Tokyo, Japan), the total RNA was reverse‐transcribed to cDNA using ReverTra Ace (TRT‐101, TOYOBO, Osaka, Japan), random hexamers, and oligo (dT) 20 primers. Transcribed cDNA was used for quantitative PCR analysis using TB Green Premix *Ex Taq* or *Ex Taq* II DNA polymerase (RR420 or RR820, Takara Bio, Shiga, Japan) at an annealing temperature of 58–60°C using a Thermal Cycler Dice TP850 (Takara Bio). Specific primers used for target genes were as follows: *Atf‐4*, sense 5′‐AATGGCTGGCTATGGATGGG‐3′ and antisense 5′‐TGTCTGAGGGGGCTCCTTATTAG‐3′; Spliced *Xbp1*, sense 5′‐GAGTCCGCAGCAGGTG‐3′ and antisense 5′‐TCCCATGGATTCTGACGC‐3′; *Bip*/*Grp78*, sense 5′‐CCTGCGTCGGTGTATTCAAG‐3′ and antisense 5′‐AAGGGTCATTCCAAGTGCG‐3′; *Chop*, sense 5′‐TGGAAGCCTGGTATGAGGATCTG‐3′ and antisense 5′‐GAGGTGCTTGTGACCTCTGCTG‐3′; *Caspase‐12*, sense 5′‐AAAGGGATAGCCACTGCTGAT‐3′ and antisense 5′‐CCACTCTTGCCTACCTTCC‐3′; and *β‐Actin*, sense: 5′‐TGTCACCAACTGGGACGATA‐3′ and 5′‐GGGGTGTTGAAGGTCTCAAA‐3′. The relative expression levels of the target genes were calculated using the standard curve method with Ct values. Specificity of each PCR product was confirmed by electrophoresis and dissociation curve analysis.

### 2.8 Statistical analysis

Data were analyzed using Student's *t*‐test or one‐way analysis of variance (ANOVA) with Tukey–Kramer's post‐hoc test using JMP statistical software version 8.0.1 (SAS Institute, Cary, NC, USA; RRID:SCR_014242). Data are shown as the mean ± standard deviation (SD), and *p*‐values less than 0.05 were defined as statistically significant.

## 3 RESULTS

### 3.1 Insulin suppresses apoptosis triggered by ER stress

**FIGURE 1:**
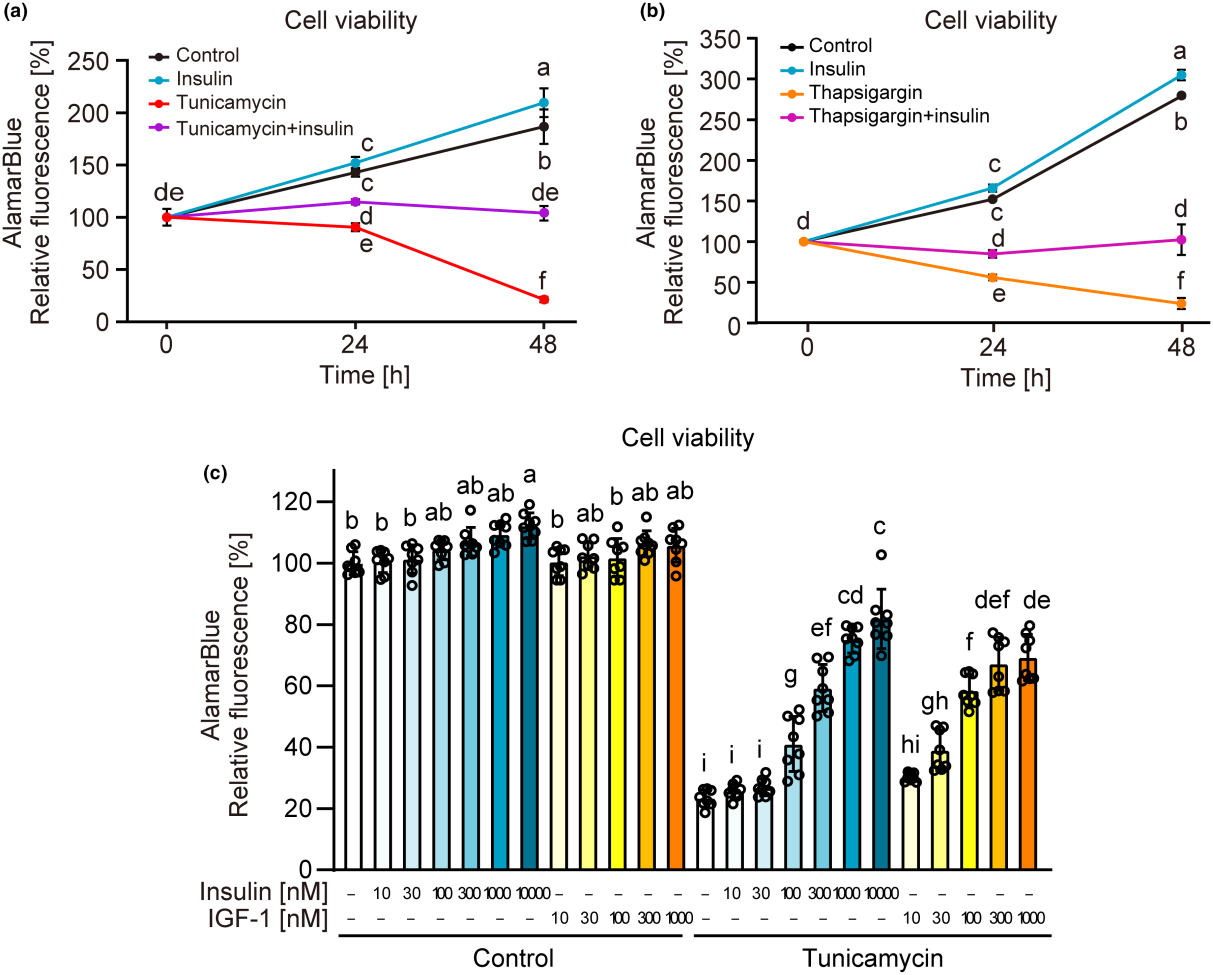
The cytoprotective effect of insulin on ER stress‐induced cell death in INS‐1 β‐cells. INS‐1 cells were incubated with tunicamycin or thapsigargin in the presence or absence of insulin for 48 h, followed by determination of cell viability using AlamarBlue dye. (a, b) Time‐dependent effects of insulin (1 μM) and tumicamycin (3 μM, for a) or thapsigargin (30 nM, for b) on cell viability. (c) The effects of insulin or IGF‐1 on tunicamycin (3 μM)‐induced loss of cell viability. Graph is shown as mean ± SD (*n* = 4 or 8). Different letters indicate statistically significant differences (*p* < 0.05, ANOVA, Tukey–Kramer's test).

Time‐dependent increase in INS‐1 cell viability was abolished by treatment with tunicamycin for 24 h, and cell viability decreased on treatment with tunicamycin for 48 h (Figure 1a). Insulin treatment (1 μM) slightly increased cell viability under non‐stress conditions. Moreover, it markedly prevented the decrease in cell viability caused by tunicamycin. Similar results were obtained when thapsigargin was used as an ER stress inducer instead of tunicamycin (Figure 1b). IGF‐1 suppresses ER stress‐induced loss of viability in β‐cells (Srinivasan et al., 2005). Recently, the physiological concentrations of insulin and IGF‐1 surrounding β‐cells were estimated to be 1 μM and 30 nM, respectively (Jansson et al., 2016; Karna et al., 2002; Thissen et al., 1994). The physiological importance of insulin and IGF‐1 on β‐cell viability under ER stress was then assessed by evaluating the effective dose. As shown in Figure 1c, both insulin and IGF‐1 exerted dose‐dependent cytoprotective effects against tunicamycin. At a low concentration range (e.g., 30 and 100 nM), cytoprotection by IGF‐1 was higher than that by insulin when compared at the same concentrations. However, at each physiological concentration surrounding β‐cells, insulin (1 μM) exerted substantially higher cytoprotection than IGF‐1 (30 nM).

**FIGURE 2:**
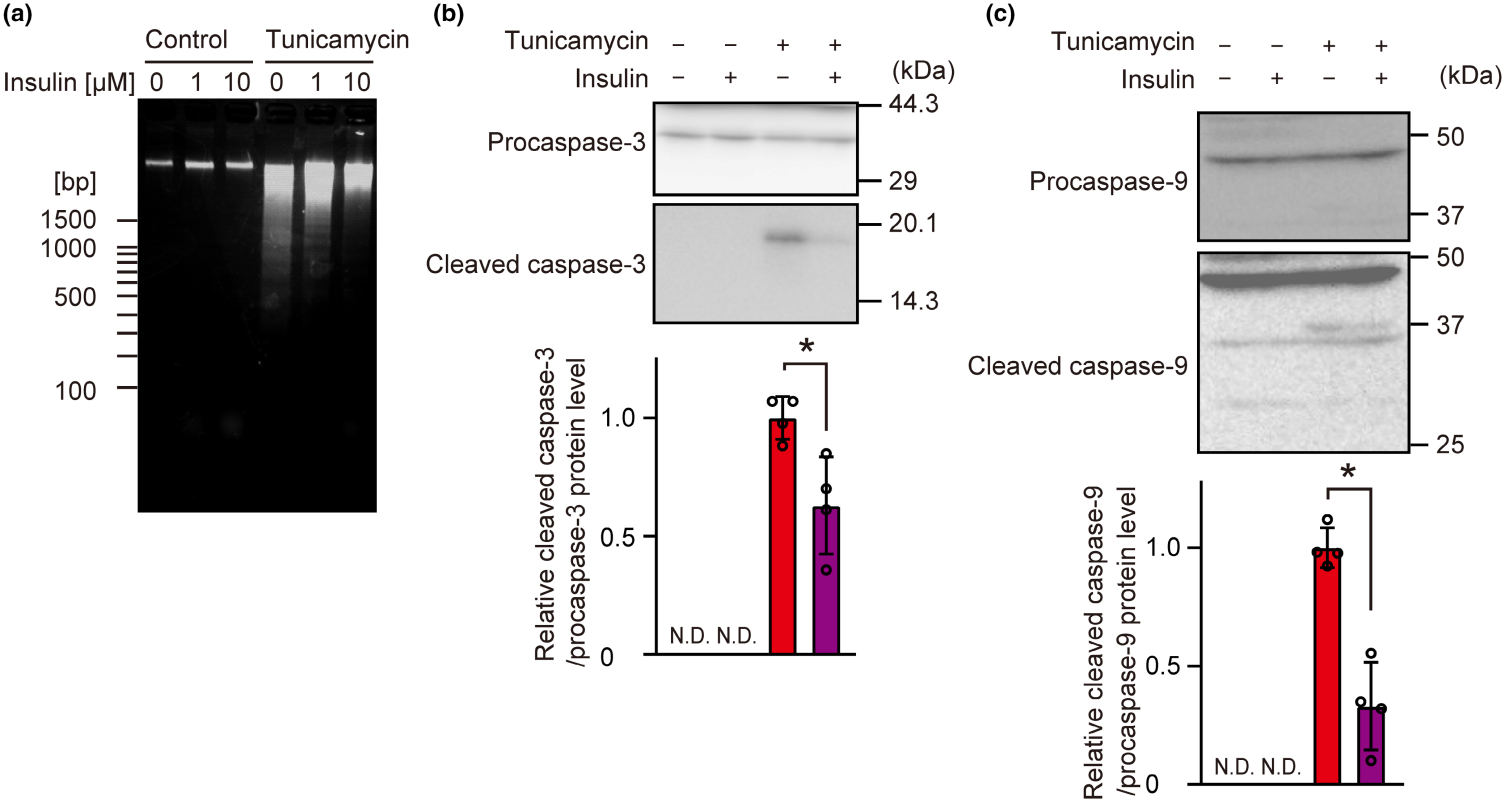
Antiapoptotic influence of insulin on ER stress‐induced cell death in INS‐1 β‐cells. INS‐1 cells were incubated in the presence of insulin (1 μM) and tunicamycin (3 μM) for 48 h (a), 36 h (b, c). (a) DNA laddering was evaluated. The results were representative of three independent experiments. (b, c) Cell lysates were analyzed by western blotting with anti‐cleaved caspase‐3 and total caspase‐3 (b) or with anti‐cleaved caspase‐9 and total caspase‐9 (c) antibodies. Representative blots and densitometry analysis are shown. Graph is shown as mean ± SD (*n* = 4). Asterisk indicates statistically significant differences (*p* < 0.05, Student's *t*‐test). N.D, not detected.

We also assessed whether the cytoprotective action of insulin was due to suppression of apoptosis. DNA laddering was induced by tunicamycin and suppressed by insulin (Figure 2a). In addition, the active forms of cleaved caspase‐3 and caspase‐9 were induced by tunicamycin and suppressed by insulin (Figure 2b,c). These results highlighted the antiapoptotic function of insulin in its cytoprotective activity under ER stress conditions.

### 3.2 Effects of insulin on ER stress‐induced expression of unfolded protein response genes

**FIGURE 3:**
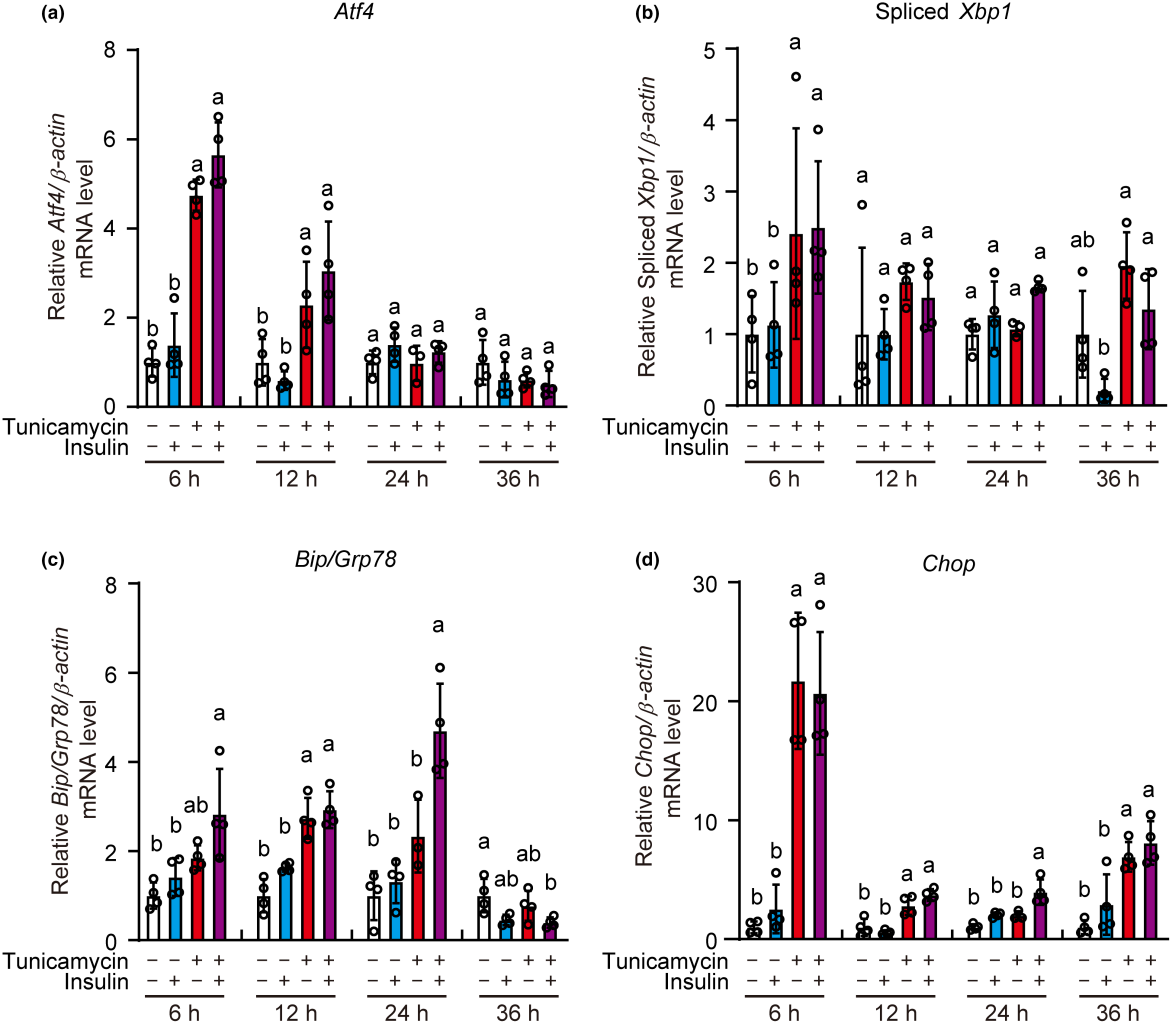
No suppressive action of insulin in ER stress‐induced increase of unfolded protein response genes. INS‐1 cells were incubated in the presence and absence of insulin (1 μM) and/or tunicamycin (3 μM) for 6, 12, 24, and 36 h. Expressions of the unfolded protein response genes (a) *Atf4*, (b) Spliced *Xbp1*, (c) *Bip*/*Grp78*, and (d) *Chop* were determined by qRT‐PCR. The expression of *β‐Actin* was used as internal control. Graph is shown mean ± SD (*n* = 4). Different letters at each time point indicate statistically significant differences at the same time points (*p* < 0.05, ANOVA, Tukey–Kramer's test).

To assess the effects of insulin on the unfolded protein response, the expression levels of the unfolded protein response genes were determined by qRT‐PCR. The expression of *Atf4*, a downstream target of PERK, was induced by tunicamycin 6 and 12 h after stimulation, whereas insulin had no significant effects on *Atf4* expression (Figure 3a). *Xbp1* is spliced by IRE1, thus, spliced *Xbp1* is a marker of IRE1 activation. The expression of spliced *Xbp1* was induced by tunicamycin after 6 h of treatment, however, was not affected by insulin (Figure 3b). The expression of *Bip/Grp78*, a target of ATF6, and *Chop*, a downstream target of ATF4, XBP1, and ATF6, increased after 6, 12, and/or 36 h of tunicamycin treatment and was not affected by insulin treatment, however, expression increased 24 h after co‐treatment with insulin (Figure 3c,d). Our results indicate that insulin had no significant effects on gene expression of unfolded protein response genes in the absence of ER stress, whereas it could slightly enhance *Bip/Grp78* and *Chop* expression under ER stress.

### 3.3 Reduction of ER stress‐induced apoptosis by insulin is independent of the mitochondrial intrinsic pathway but dependent on the mitochondrial membrane potential

**FIGURE 4:**
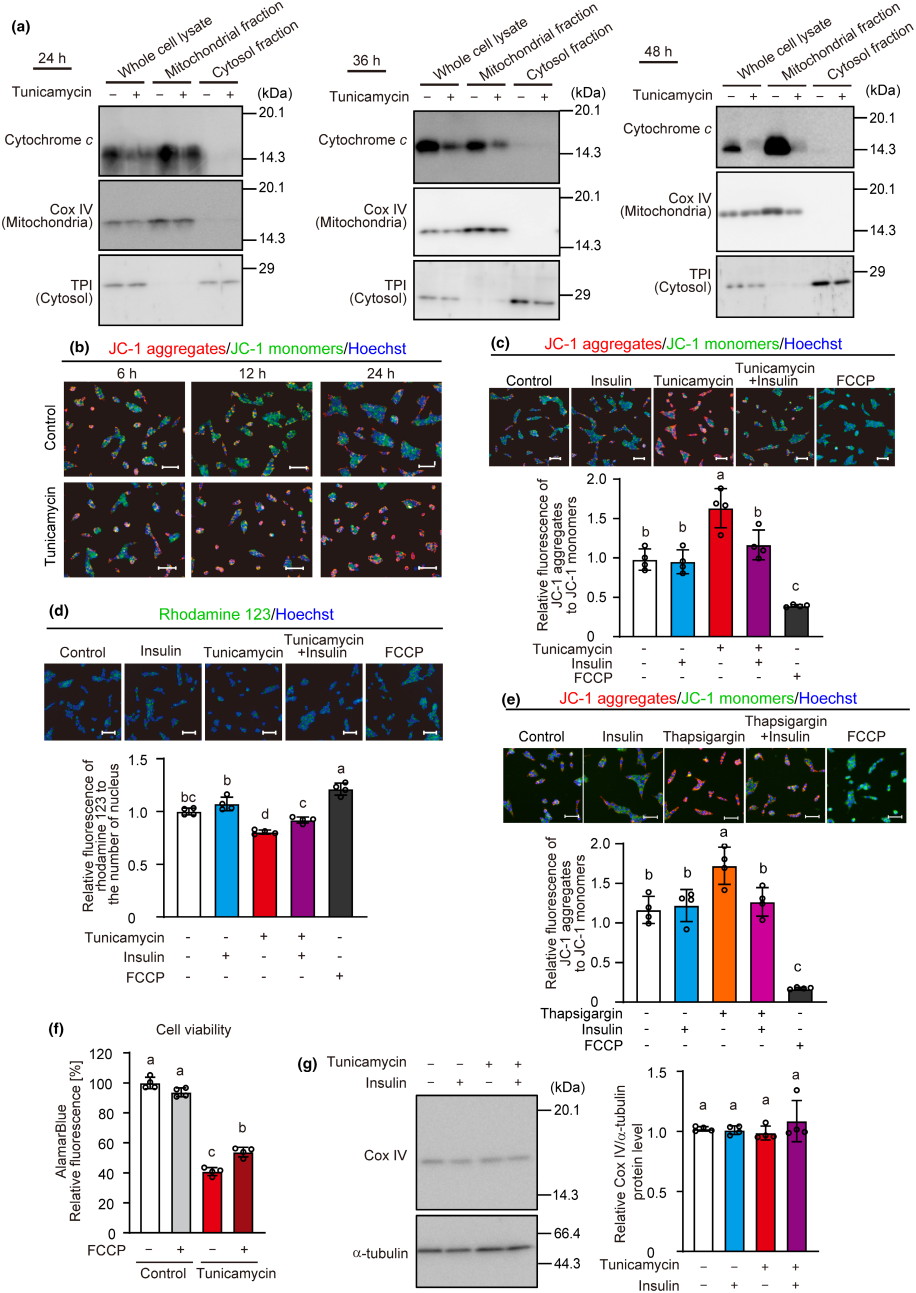
Suppression of ER stress‐induced mitochondrial membrane hyperpolarization by insulin. INS‐1 cells were incubated with tunicamycin (3 μM) and insulin (1 μM) for the specified time (a, b), 12 h (c–e), and 24 h (g). (a) The cell lysates were analyzed by western blotting with anti‐cytochrome *c*, anti‐Cox IV (mitochondria marker), and anti‐triosephosphate isomerase (TPI, cytosol marker). (b–e) The cells were incubated with JC‐1 dye (b, c, e) or rhodamine 123 (d), Hoechst 33342, and FCCP (20 μM) for 30 min and were observed using fluorescent microscopy (b, c, e red: JC‐1 aggregates, green: JC‐1 monomer, blue: Hoechst 33342; d, green: rhodamine 123, blue: Hoechst 33342, scale bar = 50 μm). Ratio of fluorescence of JC‐1 aggregates to JC‐1 monomer (c, e) or intensity of rhodamine 123 fluorescence in each cell (e) was determined. (f) INS‐1 cells were incubated with tunicamycin (3 μM) and FCCP (2 μM) for 36 h, and cell viability was determined using AlamarBlue dye. (g) Cell lysates were analyzed by western blotting with anti‐Cox IV and α‐tubulin antibodies. Subsequently, band intensity was determined. Graph is shown as mean ± SD (*n* = 4). Different letters indicate statistically significant differences (*p* < 0.05, ANOVA, Tukey–Kramer's test).

To examine the effects of ER stress on the mitochondrial apoptotic pathway, the release of cytochrome *c* from the mitochondria was evaluated by subcellular fractionation. Even after 24, 36, and 48 h of tunicamycin treatment the cytochrome *c* was not released into the cytosol (Figure 4a). Subsequently, we examined mitochondrial membrane polarization, which is involved in the induction of apoptosis, using JC‐1 dye. Tunicamycin treatment increased the fluorescence ratio of the aggregated JC‐1 dye (red) to the monomer JC‐1 (green) in a time‐dependent manner (Figure 4b). This indicates that ER stress caused mitochondrial hyperpolarization. This hyperpolarization was attenuated in the presence of insulin (Figure 4c). As a control, we used FCCP, a mitochondrial oxidative phosphorylation uncoupler, which decreased the ratio of JC‐1 aggregates to JC‐1 monomers, confirming this approach. Rhodamine 123 was used in further validation. FCCP increased the rhodamine 123 fluorescence per cell, whereas tunicamycin decreased rhodamine 123 fluorescence, which was diminished by insulin treatment (Figure 4d). Insulin also attenuated the thapsigargin‐induced mitochondrial hyperpolarization (Figure 4e). FCCP at 20 μM remarkably decreased mitochondrial polarization and did not affect cell viability in short‐time incubation for determining mitochondrial polarization (Figure 4c–e), whereas it decreased cell viability after a long‐time treatment (data not shown). FCCP at 2 μM marginally affected cell viability but effectively prevented ER stress‐induced loss of cell viability (Figure 4f). Tunicamycin and insulin had no effect on mitochondrial content assessed by quantifying the mitochondria‐specific protein, COX IV (Figure 4g). Taken together, these results suggest that insulin suppresses cytotoxicity, partly by suppressing mitochondrial hyperpolarization.

### 3.4 Cytoprotection by insulin against ER stress is independent of its anti‐oxidative activity

**FIGURE 5:**
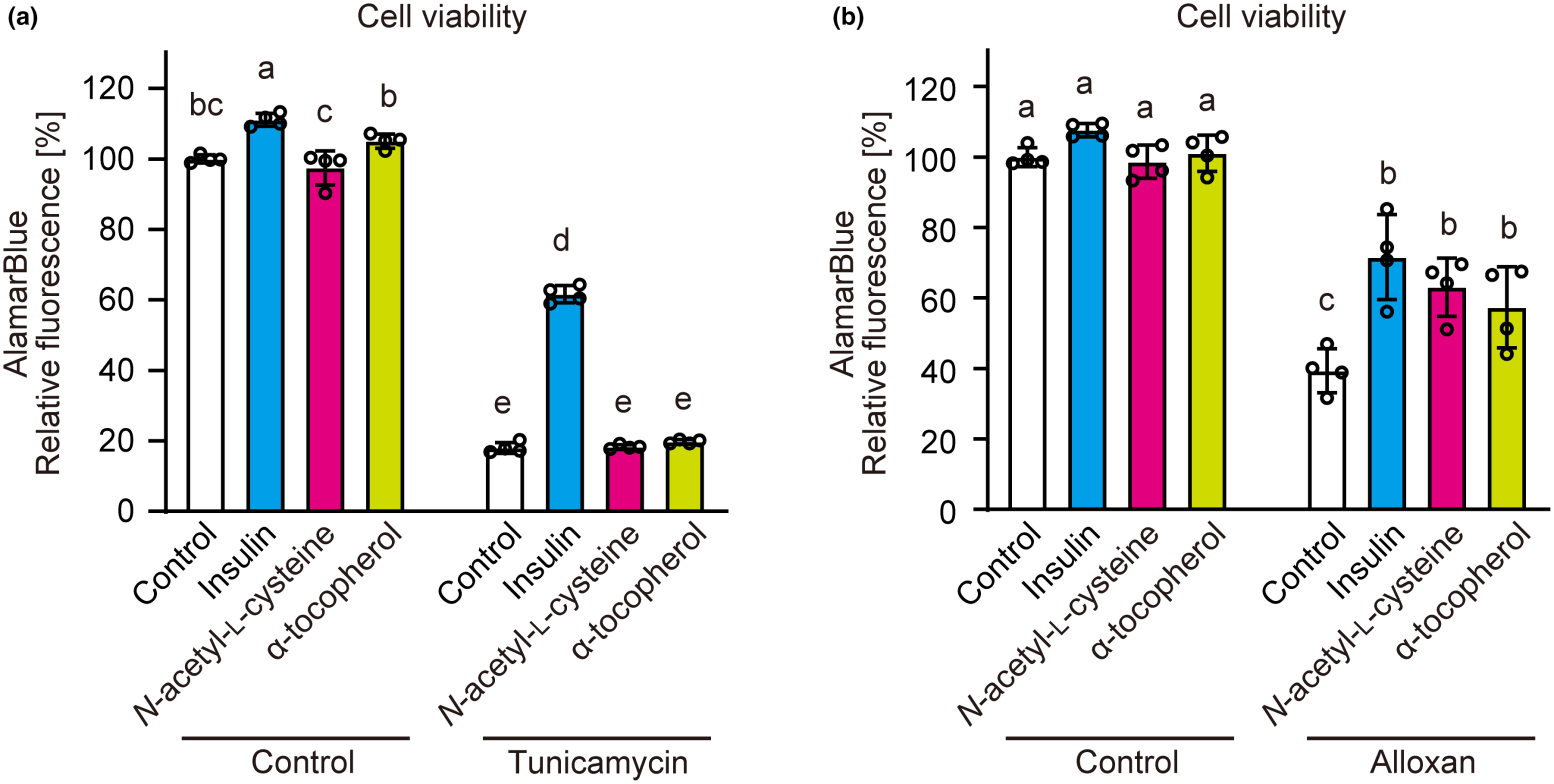
Amelioration of ER stress‐induced loss of cell viability by insulin independent of its anti‐oxidative activity. INS‐1 cells that were pretreated with insulin (1 μM), *N*‐acetylcysteine (500 μM), or α‐tocopherol (vitamin E, 20 μM) for 30 min were incubated with 3 μM tunicamycin for 48 h (a) or 5 mM alloxan for 24 h (b), and cell viability was determined using AlamarBlue dye. Graph is shown as mean ± SD (*n* = 4). Different letters indicate statistically significant differences (*p* < 0.05, ANOVA, Tukey–Kramer's test).

Along with ER stress, oxidative stress is a major stress in β‐cells and is also associated with mitochondrial dysfunction. By comparing antioxidants, we examined whether insulin exerted cytoprotective effects by decreasing oxidative stress. As a result, unlike insulin, the antioxidants *N*‐acetyl‐l‐cysteine and α‐tocopherol (vitamin E) did not affect the loss of cell viability by tunicamycin (Figure 5a). In contrast, in addition to insulin, *N*‐acetyl‐l‐cysteine and α‐tocopherol suppressed the decrease in cell viability induced by the oxidative stress inducer alloxan (Figure 5b). Thus, protection against ER stress‐induced apoptosis is independent of the anti‐oxidative activity of insulin.

### 3.5 Involvement of caspase‐12 in ER stress‐induced insulin‐suppressed apoptosis

**FIGURE 6:**
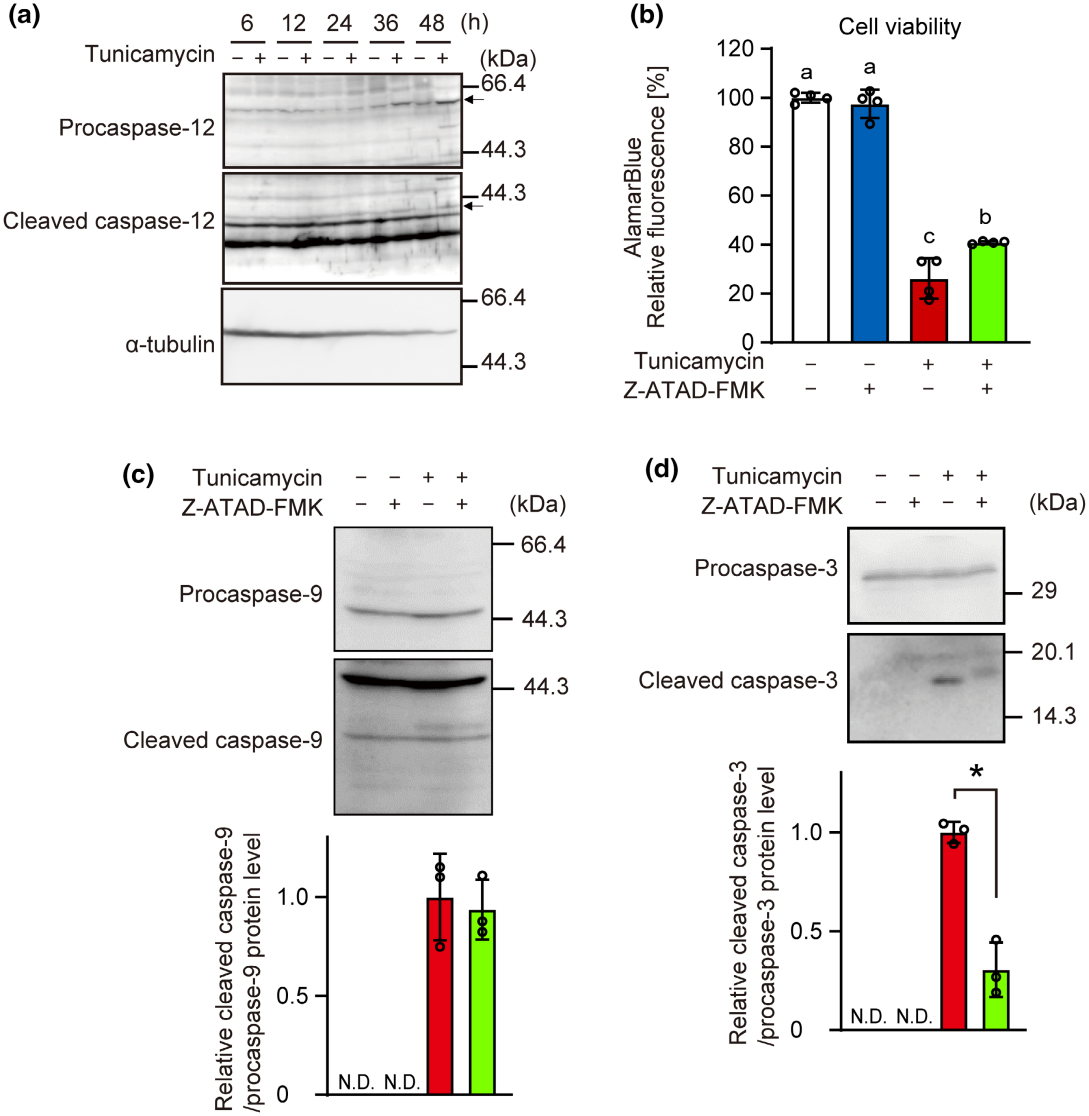
Involvement of caspase‐12 in ER stress‐induced apoptosis in INS‐1 cells. (a) INS‐1 cells were incubated with tunicamycin (3 μM) for indicated time. The cell lysates were analyzed with western blotting with anti‐caspase‐12 and α‐tubulin antibodies. (b–d) INS‐1 cells that had pretreated with Z‐ATAD‐FMK (50 μM) for 1 h were incubated with 3 μM tunicamycin for 36 h. Cell viability was determined using AlamarBlue dye (b). The cell lysates were analyzed with western blotting with anti‐caspase‐9, anti‐caspase‐3, and α‐tubulin antibodies. Subsequently, band intensity was determined (c, d). Graph is shown as mean ± SD (*n* = 3 or 4). Different letters indicate statistically significant differences (*p* < 0.05, ANOVA, Tukey–Kramer's test). Asterisk indicates statistically significant differences (*p* < 0.05, Student's *t*‐test). N.D, not detected.

Tunicamycin treatment for increased the procaspase‐12 and caspase‐12 levels in INS‐1 cells (Figure 6a). Moreover, the caspase‐12 inhibitor Z‐ATAD‐FMK exerted cytoprotective effects against tunicamycin‐induced ER stress (Figure 6b). ER stress‐induced increase in the level of cleaved caspase‐3, but not cleaved caspase‐9, was reduced in the presence of caspase‐12 inhibitor (Figure 6c,d).

**FIGURE 7:**
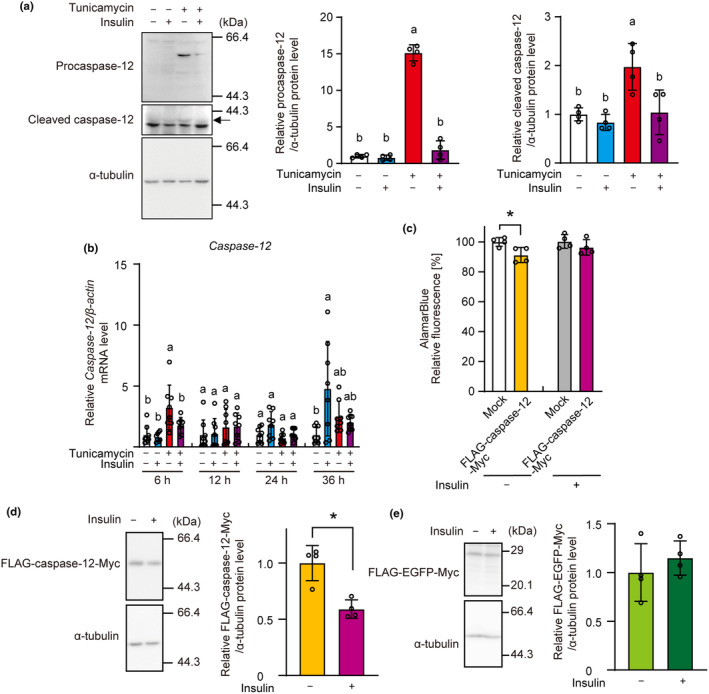
Reduction of ER stress‐induced caspase‐12 protein expression by insulin. (a, b) INS‐1 cells were incubated with tunicamycin (3 μM) and insulin (1 μM) for 48 h (a), or indicated time (b). Cell lysates were analyzed by western blotting with anti‐caspase‐12 and α‐tubulin antibodies. Subsequently, band intensity was determined. The expressions of *Caspase‐12* were determined by qRT‐PCR. The expression of *β‐Actin* was used as internal control. (c) INS‐1 cells were transfected with mock or p3 × Flag‐caspase‐12‐Myc vector and treated with insulin for 48 h. Cell viability was determined using AlamarBlue dye. (d, e) INS‐1 cells were transfected with p3 × FLAG‐caspase‐12‐Myc (d) or p3 × FLAG‐EGFP‐Myc (e) vector and treated with insulin for 48 h. The cell lysates were analyzed with western blotting with anti‐Flag and α‐tubulin antibodies. Subsequently, the band intensity was determined. Graph is shown as mean ± SD (*n* = 4 or 8). Different letters indicate statistically significant differences (*p* < 0.05, ANOVA, Tukey–Kramer's test). Asterisk indicates statistically significant differences (*p* < 0.05, Student's *t*‐test).

Insulin suppressed the tunicamycin‐induced increases in level of procaspase‐12 and cleaved caspase‐12 (Figure 7a). Although *Caspase‐12* increases was induced by tunicamycin and suppressed by insulin after 6 h of treatment, tunicamycin did not affect its expression after 24 or 36 h of treatment (Figure 7b). Overexpression of caspase‐12 reduced cell viability, which was not observed when the cells were incubated with insulin (Figure 7c). Exogenously overexpressed caspase‐12 protein levels, but not those of EGFP (negative control), also decreased when the cells were incubated with insulin (Figure 7d,e). These results indicate that ER stress occurs through an increase in caspase‐12 and that insulin suppresses this increase in β‐cells.

## 4 DISCUSSION

IGF‐1 protects ER stress‐induced apoptosis in β‐cells (Srinivasan et al., 2005), however, the detailed mechanisms remain unclear. In this study, we show that insulin also protected β‐cells from ER stress‐induced apoptosis. Notably, the cytoprotective potential of insulin was higher than that of IGF‐1 at physiologically relevant concentrations. Insulin did not decrease ER stress marker gene expression (e.g., *Atf4*, spliced *Xbp1s*, *Bip/Grp78*, and *Chop*), but rather increased *Bip/Grp78* and *Chop* expression. Because increased *Bip/Grp78* contributes to ER stress alleviation, the antiapoptotic action of insulin may be partly due to the promotion of an adaptive unfolded protein response that restores cellular homeostasis. Mitochondrial hyperpolarization is involved in ER stress‐induced apoptosis. Insulin reduced ER stress‐induced apoptosis by decreasing mitochondrial hyperpolarization and caspase‐12 protein levels. The diabetic model Akita mice harboring a *Ins2* ^*C96Y*^ mutation and unable to produce sufficient insulin, lead to excess ER stress and β‐cell loss due to increased apoptosis (Ron, 2002). Enhanced ER stress and reduction in a β‐cell subset with enhanced insulin secretion was observed in T2DM patients (Rubio‐Navarro et al., 2023). Our results revealed that defect in insulin secretion was responsible for the decrease in β‐cell mass induced by excess ER stress.

Caspase‐12 is a major mediator of ER stress‐induced apoptosis, and cleavage of caspase‐12 occurs in response to ER stress but not in the presence of other apoptotic stimuli (Rao et al., 2004). Caspase‐12 protein levels increased after 36 h of tunicamycin stimulation, whereas increase in *Caspase‐12* mRNA was observed only after 6 h of tunicamycin stimulation. Although it may depend on cell type, our results showing that caspase‐12 undergoes post‐translational regulation under ER stress were similar to the results of PC12 cells by Mao et al. (2006), but differ from the results of L6 myoblasts by Zhang et al. (2016) who reported that an increase in caspase‐12 protein is associated with *Caspase‐12* mRNA levels. Moreover, insulin reduced the tunicamycin‐induced increase in procaspase‐12 and cleaved caspase‐12 levels. Although insulin also attenuated *Caspase‐12* mRNA after 6 h of tunicamycin stimulation, it had no effect thereafter. Moreover, the overexpression of caspase‐12 caused apoptosis, and insulin reduced the overexpressed caspase‐12 protein levels. Thus, our results indicate that insulin protects against ER stress‐induced apoptosis by reducing caspase‐12 levels at the post‐translational stage.

ER stress progressively induced mitochondrial hyperactivation in INS‐1 cells. Although this differs from the general apoptosis model of the mitochondrial intrinsic pathway, in which mitochondrial depolarization is associated with apoptosis (Bock & Tait, 2020), cytochrome *c* can be released from the mitochondria into cytoplasm before mitochondrial membrane depolarization (Yang et al., 1997). Hyperpolarization of the mitochondrial membrane can also induce cytochrome *c* release (Giovannini et al., 2002; Sánchez‐Alcázar et al., 2000), leading to apoptosis. The mechanism underlying mitochondrial hyperpolarization leading to apoptosis in β‐cells remains unclear. However, during apoptosis, cytochrome *c* release was not observed during 24–48 h of tunicamycin treatment. Luciani et al. (2009) reported that mitochondrial membrane hyperactivation occurred under ER stress in MIN6 β‐cells. Kawai et al. (2006) reported that cytochrome *c* expression is suppressed under ER stress, suggesting the occurrence of apoptosis independent of the mitochondrial pathway. ER stress‐induced mitochondrial hyperpolarization is reduced by insulin treatment. In addition, FCCP (2 μM) exerts cytoprotective effects against ER stress. This concentration could also prevent mitochondrial hyperpolarization under ER stress conditions but still permit ATP production. Collectively, mitochondrial hyperpolarization under ER stress is likely involved in the ER stress‐induced and insulin‐mediated decrease in apoptosis.

Both insulin and IGF‐1 are thought to contribute to the development and maintenance of β‐cells (Ueki et al., 2006). However, the major factor for β‐cell mass control has not yet been clarified. Recently, intra‐islet extracellular insulin concentrations were estimated to be approximately 1 μM (Jansson et al., 2016), whereas both the pancreas and blood IGF‐1 concentrations were found to be approximately 30 nM (Karna et al., 2002; Thissen et al., 1994). IGF‐1 is predominantly synthesized and secreted by the liver, and pancreatic IGF‐1 expression is downregulated after weaning (Calvo et al., 1997). In addition, RNA sequence analyzes showed that the RPKM value of IGF‐1 mRNA was 1/10^4^–1/10^6^ of insulin mRNA in the human islets (Eizirik et al., 2012; Segerstolpe et al., 2016). Srinivasan et al. (2005) showed that IGF‐1 protects β‐cells from ER stress‐induced apoptosis. Our results showed that both insulin and IGF‐1 prevented ER stress‐induced cell loss in a dose‐dependent manner. Moreover, the cytoprotective effects of 1 μM insulin against ER stress was more potent than that of 50 nM IGF‐1. A recent study showed that the inceptor counteracts insulin sensing in β‐cells (Ansarullah et al., 2021). Collectively, insulin is more physiologically important than IGF‐1 in terms of its cytoprotective effects under physiological and pathophysiological conditions.

Unfolded protein response genes increase under ER stress by regulating three major pathways (i.e., PERK, IRE‐1, and ATF6) (Hetz, 2012). The marker genes of all three major pathways were not attenuated, but rather promoted by insulin. Similarly, IGF‐1 had no effect on ER stress‐induced *Chop* expression in MIN6 β‐cells (Srinivasan et al., 2005), but increased ER‐stress‐induced *Chop* expression in NIH3T3 fibroblasts (Novosyadlyy et al., 2008). Pancreatic β‐cells are easily affected by oxidative stress (Hasnain et al., 2016), and ER stress could induce oxidative stress (Cao & Kaufman, 2014). Antioxidants, such as *N*‐acetyl‐l‐cysteine and vitamin E, suppressed oxidative stress‐induced cell death but did not provide cytoprotection under ER stress. By contrast, insulin suppressed both oxidative stress‐ and ER stress‐induced cell death. Therefore, these results suggest that insulin reduces ER stress‐induced apoptosis without suppressing ER stress and is independent of its anti‐oxidative activity.

The caspase‐12 inhibitor Z‐ATAD‐FMK suppressed the tunicamycin‐induced loss of cell viability and caspase‐3 cleavage. In MEF cells, it is proposed that ER‐stress activates caspase‐12, caspase‐9, and caspase‐3 pathway in mentioned order (Rao et al., 2002), whereas caspase‐9 is a substrate of caspase‐12 and needs to be further studied (Lee et al., 2010). In this study, Z‐ATAD‐FMK decreased the tunicamycin‐induced increase in the cleavage of caspase‐3 but not caspase‐9, whereas it decreased the levels of both caspase‐3 and caspase‐9 in PC‐12 cells (Mao et al., 2006). As the overexpression of caspase‐12 activates caspase‐3 (Hitomi et al., 2004), caspase‐12 plays a key role in ER stress‐induced apoptosis.

Prevention of ER stress‐induced β‐cell apoptosis is important for prevention of T2DM. In this study, insulin was found to decrease ER stress‐induced β‐cell apoptosis, presumably independent from decreasing ER stress. Insulin also regulated ER stress‐induced mitochondrial hyperpolarization and caspase‐12 levels. At physiological concentrations, insulin seems to play a more important role in β‐cell mass regulation than IGF‐1. A limitation of this study is that all experiments were performed using INS‐1 β‐cells. Present results warrant further investigation using isolated islets and animal models. Prolonged insulin action could cause β‐cell death (Bucris et al., 2016; Rachdaoui et al., 2019), but switching of insulin action as an antiapoptotic factor to pro‐apoptotic factor also needs to be clarified in future studies. Our findings shed light on the mechanism whereby defects in insulin secretion cause β‐cell loss.

## AUTHOR CONTRIBUTIONS

N.H. conceived research; N.M., K.N., and N.H. designed research; N.M. and K.N. performed experiments; N.M., K.N., N.H., T.K., E.Y., H.I., and R.Y. analyzed data and interpreted results of experiments; N.M. and K.N. prepared figures; N.M. and N.H. drafted manuscript; N.H. edited and revised manuscript; N.M., K.N., N.H., T.K., E.Y., H.I., and R.Y. approved final version of manuscript.

## FUNDING INFORMATION

This work was supported by the Japan Society for the Promotion Science (JSPS) KAKENHI (Grant Number 21K19093, 22H02289, to N.H.), Thomas J. Beatson, Jr. Foundation Grant (#2022‐006, to E.Y.), National Institute of Diabetes And Digestive And Kidney Diseases (NIDDK, R01DK136888, to E.Y.), and JDRF (5‐CDA‐2022‐1178‐A‐N, to E.Y.).

## CONFLICT OF INTEREST STATEMENT

The authors have no conflicts of interest to declare.

## ETHICS STATEMENT

Not applicable.

## Data Availability

The data generated during this study are available from the corresponding author (N.H.) upon request.
